# Supplementary material for: Prognostic Values of Ferroptosis-Related Proteins ACSL4, SLC7A11, and CHAC1 in Cholangiocarcinoma
Source: Biomedicines. 2024 Sep 13;12(9):2091. doi: 10.3390/biomedicines12092091 (PMC11428419; doi:10.3390/biomedicines12092091)
Supplement: Supplementary file 1 [file biomedicines-12-02091-s001.zip › biomedicines-3173164-supplementary.pdf]

Supplementary Table S1 Correlation between ACSL4, SLC7A11, and CHAC1 expression and clinicopathological data of CCA patients

Characteristics	ACSL4 expression			SLC7A11 expression			CHAC1 expression		
	Low n, (%)	High n, (%)	*p*-value	Low n, (%)	High n, (%)	*p*-value	Low n, (%)	High n, (%)	*p*-value
Gender			0.29			0.41			0.41
Female	22 (43.1%)	29 (56.9%)		28 (54.9%)	23 (45.1%)		22 (43.1%)	29 (56.9%)	
Male	45 (52.3%)	41 (47.7%)		41 (47.7%)	45 (52.3%)		31 (36.0%)	55 (64.0%)	
Age (median, years)			0.65			0.54			0.93
< 60	30 (46.9%)	34 (53.1%)		34 (53.1%)	30 (46.9%)		25 (39.1%)	39 (60.9%)	
≥ 60	37 (50.7%)	36 (49.3%)		35 (47.9%)	38 (52.1%)		28 (38.4%)	45 (61.6%)	
Location of tumor			0.05			0.16			0.67
Intrahepatic	53 (54.1%)	45 (45.9%)		53 (54.1%)	45 (45.9%)		39 (39.8%)	59 (60.2%)	
Extrahepatic	14 (35.9%)	25 (64.1%)		16 (41.0%)	23 (59.0%)		14 (35.9%)	25 (64.1%)	
Tumor growth type			0.06			**0.01**			0.11
Intraductal type	16 (61.5%)	10 (38.5%)		14 (53.8%)	12 (46.2%)		9 (34.6%)	17 (65.4%)	
Mass forming type	21 (58.3%)	15 (41.7%)		26 (72.2%)	10 (27.8%)		20 (55.6%)	16 (44.4%)	
Mixed type	25 (39.1%)	39 (60.9%)		25 (39.1%)	39 (60.9%)		23 (35.9%)	41 (64.1%)	
Cell type			0.94			0.92			0.20
papillary	36 (48.6%)	38 (51.4%)		37 (50.0%)	37 (50.0%)		25 (33.8%)	49 (66.2%)	
non-papillary	31 (49.2%)	32 (50.8%)		32 (50.8%)	31 (49.2%)		28 (44.4%)	35 (55.6%)	
Lymph node metastasis			0.81			0.86			0.93
Yes	35 (47.9%)	38 (52.1%)		36 (49.3%)	37 (50.7%)		28 (38.4%)	45 (61.6%)	
No	32 (50.0%)	32 (50.0%)		33 (51.6%)	31 (48.4%)		25 (39.1%)	39 (60.9%)	
Distance metastasis			0.38			0.35			0.33
Yes	4 (36.4%)	7 (63.6%)		7 (63.6%)	4 (36.4%)		6 (54.5%)	5 (45.5%)	
No	63 (50.0%)	63 (50.0%)		62 (49.2%)	64 (50.8%)		47 (37.3%)	79 (62.7%)	
TMN Stage			0.99			0.33			0.87
I-II	22 (48.9%)	23 (51.1%)		20 (44.4%)	25 (55.6%)		17 (37.8%)	28 (62.2%)	
III-IV	45 (48.9%)	47 (51.1%)		49 (53.3%)	43 (46.7%)		36 (39.1%)	56 (60.9%)	
OV infection			0.67			0.52			0.90
Yes	50 (50.0%)	50 (50.0%)		52 (52.0%)	48 (48.0%)		39 (39.0%)	61 (61.0%)	
No	17 (45.9%)	20 (54.1%)		17 (45.9%)	20 (54.1%)		14 (37.8%)	23 (62.2%)	
Total protein (g/dL)			0.11			0.12			1.00
< 8.7	54 (46.2%)	63 (53.8%)		55 (47.0%)	62 (53.0%)		45 (38.5%)	72 (61.5%)	
≥ 8.7	6 (78.0%)	2 (25.0%)		6 (75.0%)	2 (25.0%)		3 (37.5%)	5 (62.5%)	
Globulin (g/dL)			0.90			0.31			0.90
< 3.4	27 (47.4%)	30 (52.6%)		25 (43.9%)	32 (56.1%)		21 (36.8%)	36 (63.2%)	
≥ 3.4	32 (48.5%)	34 (51.5%)		35 (53.0%)	31 (47.0%)		25 (37.9%)	41 (62.1%)	
Total bilirubin (mg/dL)			0.37			0.53			0.36
< 1.2	46 (50.0%)	46 (50.0%)		43 (46.7%)	49 (53.3%)		38 (41.3%)	54 (58.7%)	
≥ 1.2	14 (41.2%)	20 (58.8%)		18 (52.9%)	16 (47.1%)		11 (32.4%)	23 (67.6%)	

Direct bilirubin (mg/dL)			0.34			0.46			0.94
< 0.5	38 (50.7%)	37 (49.3%)		34 (45.3%)	41 (54.7%)		29 (38.7%)	46 (61.3%)	
≥ 0.5	21 (42.0%)	29 (58.0%)		26 (52.0%)	24 (48.0%)		19 (38.0%)	31 (62.0%)	
ALT (U/L)			0.17			0.80			0.12
< 33	28 (54.9%)	23 (45.1%)		24 (47.1%)	27 (52.9%)		24 (47.1%)	27 (52.9%)	
≥ 33	32 (42.7%)	43 (57.3%)		37 (49.3%)	38 (50.7%)		25 (33.3%)	50 (66.7%)	
AST (U/L)			0.17			0.19			0.55
< 40	30 (54.5%)	25 (45.5%)		23 (41.8%)	32 (58.2%)		23 (41.8%)	32 (58.2%)	
≥ 40	30 (42.3%)	41 (57.7%)		38 (53.5%)	33 (46.5%)		26 (36.6%)	45 (63.4%)	
ALP (U/L)			0.81			0.77			0.85
< 129	27 (50.0%)	27 (50.0%)		26 (48.1%)	28 (51.9%)		22 (40.7%)	32 (59.3%)	
≥ 129	33 (47.8%)	36 (52.2%)				0.41	27 (39.1%)	42 (60.9%)	

Supplementary Table S2 The univariate and multivariate analysis of clinicopathological data.

variable	Overall survival							
	univariate				multivariate			
	HR	95% CI		*p*-value	HR	95% CI		*p*-value
Age (≥60)	1.08	0.76	1.52	0.64				
Gender (Male)	0.97	0.68	1.38	0.89				
Location of tumor (Intrahepatic)	0.89	0.61	1.29	0.54				
Cell types (Papillary)	0.63	0.45	0.89	**0.01**	0.79	0.55	1.11	0.17
Tumor growth type (Mixed type)	0.93	0.59	1.48	0.78				
Lymph node metastasis (Yes)	2.31	1.61	3.30	**<0.01**	1.09	0.65	1.81	0.72
Distance metastasis (Yes)	3.04	1.61	5.73	**<0.01**	2.15	1.12	4.13	0.02
TMN Stage (III-IV)	2.87	1.94	4.25	**<0.01**	2.39	1.36	4.18	**<0.01**
OV infection (Positive)	1.18	0.80	1.72	0.39				
ACSL4 (High)	0.89	0.63	1.25	0.51				
SLC7A11 (High)	0.75	0.53	1.06	0.10				
CHAC1 (High)	0.93	0.65	1.32	0.70				
